# Supplementary figures and images for: Designing novel multiepitope mRNA vaccine targeting Hendra virus (HeV): An integrative approach utilizing immunoinformatics, reverse vaccinology, and molecular dynamics simulation
Source: PLoS One. 2024 Oct 23;19(10):e0312239. doi: 10.1371/journal.pone.0312239 (PMC11498705; doi:10.1371/journal.pone.0312239)

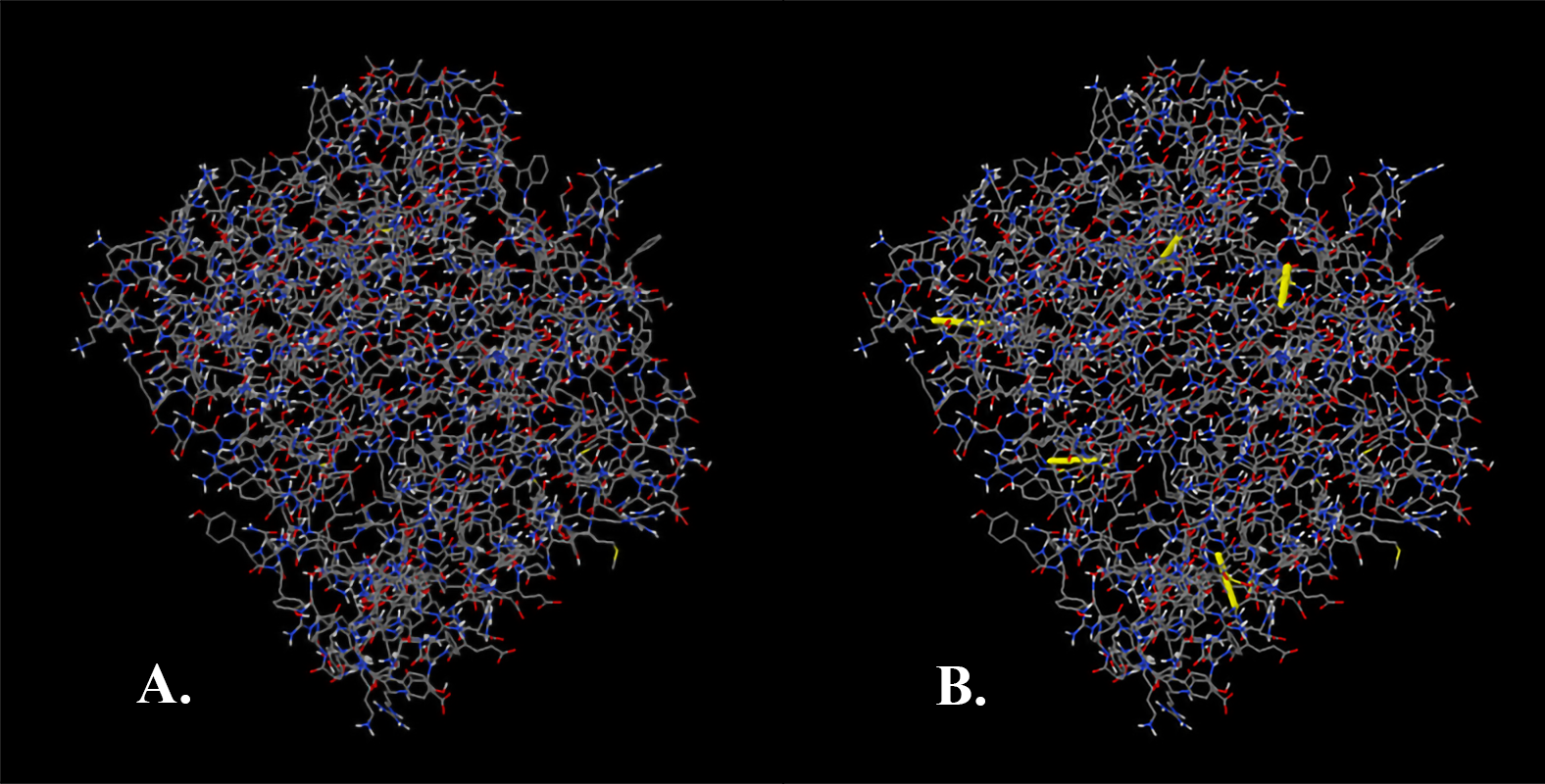

Supplement: S1 Fig — (A) The wild type; (B) The five introduced disulfide bonds are represented by yellow sticks to denote the mutant form. (TIF) [file pone.0312239.s001.tif]

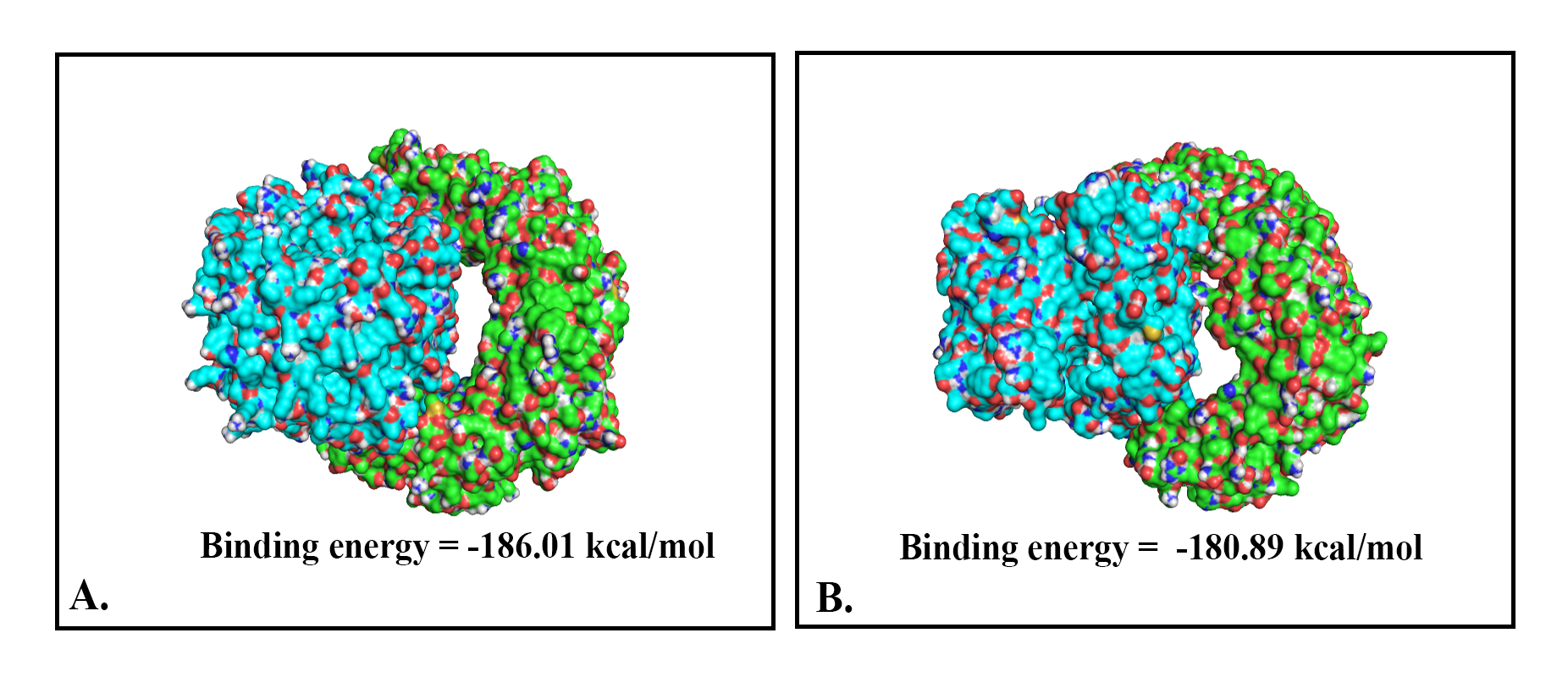

Supplement: S2 Fig — The “V-TLR-2” (A) and “V-TLR-4” (B) complexes were analyzed using MM-GBSA. The cyan ribbon represents the vaccine, and the green ribbon the receptors. (TIF) [file pone.0312239.s002.tif]

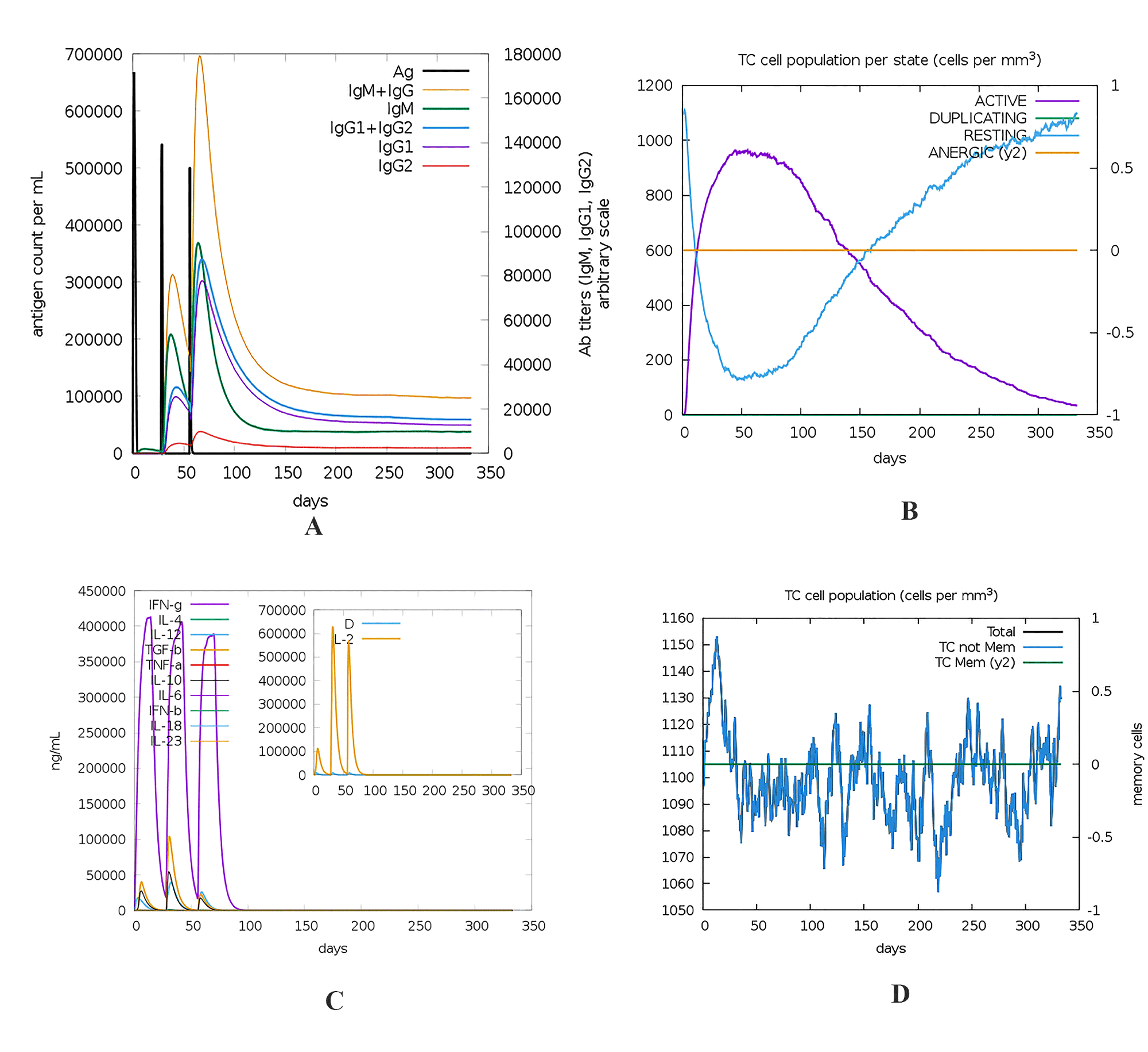

Supplement: S3 Fig — In the aftermath of three successive doses, the immune response manifests as different populations of B-cells (A), Antigen (B), TC-cells per state (C), Cytokines (D), TC cells. (TIF) [file pone.0312239.s003.tif]
