# Supplementary material for: Designing novel multiepitope mRNA vaccine targeting Hendra virus (HeV): An integrative approach utilizing immunoinformatics, reverse vaccinology, and molecular dynamics simulation
Source: PLoS One. 2024 Oct 23;19(10):e0312239. doi: 10.1371/journal.pone.0312239 (PMC11498705; doi:10.1371/journal.pone.0312239)
Supplement: S1 Table — (DOCX) [file pone.0312239.s004.docx]

**S1 Table. Pairs of vaccine residues that can form disulfide bonds are listed here.**

| **Residue 1 chain** | **Residue 1 Seq** | **Residue 1 AA** | **Residue 2 chain** | **Residue 2 Seq** | **Residue 2 AA** | **Bond Chi3** | **Energy (kcal/mol)** | **Sum B-Factors** |
| --- | --- | --- | --- | --- | --- | --- | --- | --- |
| A | 1 | MET | A | 9 | ASP | 120.76 | 4.13 | 0 |
| A | 5 | SER | A | 8 | ASP | 100.44 | 1.69 | 0 |
| A | 18 | LEU | A | 487 | LEU | 126.35 | 7.2 | 0 |
| A | 20 | ALA | A | 39 | ALA | 126.46 | 2.71 | 0 |
| A | 20 | ALA | A | 42 | THR | 100.09 | 7.04 | 0 |
| A | 75 | PHE | A | 94 | ARG | 118.24 | 3.33 | 0 |
| A | 82 | LYS | A | 90 | ALA | 115.55 | 3.66 | 0 |
| A | 87 | TYR | A | 90 | ALA | 87.8 | 1.81 | 0 |
| A | 112 | GLN | A | 120 | ALA | 104.05 | 2.89 | 0 |
| A | 117 | GLU | A | 120 | ALA | 86.82 | 0.85 | 0 |
| A | 139 | THR | A | 148 | GLY | -105.85 | 3.19 | 0 |
| A | 158 | GLU | A | 188 | ALA | 119.37 | 6.94 | 0 |
| A | 166 | ALA | A | 181 | ALA | -80.56 | 4.39 | 0 |
| A | 169 | ALA | A | 177 | PRO | 122.84 | 7.28 | 0 |
| A | 169 | ALA | A | 178 | ALA | -98.49 | 2.15 | 0 |
| A | 193 | ALA | A | 208 | GLY | -67.85 | 6.21 | 0 |
| A | 196 | VAL | A | 204 | LYS | -101.4 | 2.62 | 0 |
| A | 209 | PRO | A | 249 | VAL | 120.69 | 2.21 | 0 |
| A | 219 | THR | A | 241 | LYS | -114.48 | 5.19 | 0 |
| A | 223 | PRO | A | 238 | LYS | 77.01 | 6.33 | 0 |
| A | 226 | LYS | A | 235 | LYS | 120.92 | 4.06 | 0 |
| A | 246 | LYS | A | 265 | MET | 106.6 | 1.51 | 0 |
| A | 246 | LYS | A | 268 | LEU | 81.92 | 8.51 | 0 |
| A | 258 | ALA | A | 261 | VAL | 84.2 | 2.04 | 0 |
| A | 276 | ILE | A | 296 | THR | 122.19 | 4.98 | 0 |
| A | 281 | VAL | A | 296 | THR | 120.37 | 2.5 | 0 |
| A | 311 | ILE | A | 335 | LYS | 89.61 | 0.81 | 0 |
| A | 312 | VAL | A | 386 | ASN | 120.82 | 2.52 | 0 |
| A | 317 | GLY | A | 322 | ALA | -105.73 | 1.59 | 0 |
| A | 322 | ALA | A | 327 | ILE | 101.47 | 3.45 | 0 |
| A | 350 | ALA | A | 373 | LEU | 103.64 | 2.64 | 0 |
| A | 354 | LEU | A | 369 | ALA | 83.53 | 2.92 | 0 |
| A | 357 | GLY | A | 365 | ILE | 125.8 | 9.74 | 0 |
| A | 357 | GLY | A | 366 | ARG | 122.15 | 2.43 | 0 |
| A | 377 | TYR | A | 396 | PHE | 80.7 | 5.6 | 0 |
| A | 384 | SER | A | 392 | LEU | 77.2 | 2.37 | 0 |
| A | 389 | VAL | A | 392 | LEU | 110.14 | 2.16 | 0 |
| A | 408 | PHE | A | 436 | PHE | 111.94 | 3.78 | 0 |
| A | 412 | LEU | A | 432 | GLY | -101.83 | 0.75 | 0 |
| A | 415 | LYS | A | 428 | SER | 98.33 | 2.01 | 0 |
| A | 440 | VAL | A | 463 | CYS | -114.1 | 5.42 | 0 |
| A | 443 | LEU | A | 459 | MET | -107.42 | 2.93 | 0 |
| A | 447 | SER | A | 452 | ASN | -106.96 | 4.39 | 0 |
