# Supplementary material for: Designing novel multiepitope mRNA vaccine targeting Hendra virus (HeV): An integrative approach utilizing immunoinformatics, reverse vaccinology, and molecular dynamics simulation
Source: PLoS One. 2024 Oct 23;19(10):e0312239. doi: 10.1371/journal.pone.0312239 (PMC11498705; doi:10.1371/journal.pone.0312239)
Supplement: S2 Table — (DOCX) [file pone.0312239.s005.docx]

**S2 Table. Discotope 2.0 predicted the conformational B-cell epitopes residues of the vaccine structure.**

| **No** | **Residues** | **Number of residues** | **Scores** |
| --- | --- | --- | --- |
| 1 | A:T217, A:I220, A:L221, A:K222, A:R224, A:A225, A:K226, A:K227, A:I228, A:K229, A:S230, A:N231 | 12 | 0.745 |
| 2 | A:M1, A:A2, A:E3, A:N4, A:S5, A:N6, A:D8, A:D9, A:T44, A:D45, A:R47, A:S48, A:R49, A:E51, A:E52, A:S53, A:R54, A:A55, A:R56, A:L57, A:T58, A:K59, A:L60, A:Q61, A:E62, A:D63 | 26 | 0.737 |
| 3 | A:T27, A:V28, A:N29, A:E30, A:L31, A:I32, A:T33, A:N34, A:L35, A:R36, A:E37, A:R38, A:E40, A:E41, A:R81, A:K82, A:A83, A:A84, A:E85, A:G86, A:Y87, A:L88, A:E89, A:A90, A:A91, A:T92, A:S93, A:Y95, A:N96, A:V99, A:E100, A:E103, A:A104, A:L106, A:E107, A:L109, A:R110, A:S111, A:Q112, A:Q113, A:S114, A:F115, A:E117, A:V118, A:R121 | 45 | 0.735 |
| 4 | A:S263, A:F264, A:H267, A:Y271, A:K274, A:Y275, A:A277, A:I278, A:T279, A:K280, A:E282, A:R283, A:G284, A:K285, A:A286, A:Y287, A:Y288, A:N289, A:Q290, A:T348, A:I349, A:A351, A:Y352, A:P353, A:G355, A:V356, A:G357, A:A358, A:Y359, A:Y360, A:R362, A:K363, A:R366, A:L401, A:G402, A:Y403, A:E406, A:D407, A:D409, A:D410, A:L411, A:L412, A:E413, A:S414, A:K415, A:F416, A:E417, A:R418, A:L419, A:G420, A:S421, A:I422, A:N423, A:Y424, A:N425, A:S426, A:Y464, A:G465, A:E468, A:D469, A:E471, A:R472, A:S473, A:P474, A:K475, A:F476, A:E477, A:R478, A:I479, A:R480, A:T481, A:I482, A:A483 | 73 | 0.734 |
| 5 | A:I157, A:P160, A:K161, A:A163, A:A164, A:P165, A:A166, A:K167, A:K168, A:A169, A:A170, A:P171, A:A172, A:K173, A:K174 | 15 | 0.711 |
| 6 | A:K314, A:K315, A:R316, A:G317, A:N318, A:Y319, A:S320, A:R321, A:A322, A:Y323, A:Y324, A:T325, A:F326, A:I327, A:S328, A:F329, A:V330, A:I331, A:V332, A:E333, A:K334, A:K335, A:R336, A:G337, A:N338, A:Y341, A:E387, A:A388, A:V389, A:V390, A:K391, A:L392, A:E394, A:K395, A:R398, A:V446, A:S447, A:F448, A:N449, A:N450, A:D451, A:N452, A:S453, A:E454, A:K455, A:F456, A:E457, A:R458 | 48 | 0.7 |
| 7 | A:T139, A:A141, A:S142, A:Q143, A:T144, A:R145, A:A146, A:V147, A:G148, A:E149, A:R150, A:V196, A:K199, A:A201, A:A202, A:A203, A:K204, A:E206, A:I207, A:K210 | 20 | 0.697 |
| 8 | A:P254, A:T255, A:S256, A:W257, A:A258, A:K259 | 6 | 0.646 |
