# Supplementary material for: Designing novel multiepitope mRNA vaccine targeting Hendra virus (HeV): An integrative approach utilizing immunoinformatics, reverse vaccinology, and molecular dynamics simulation
Source: PLoS One. 2024 Oct 23;19(10):e0312239. doi: 10.1371/journal.pone.0312239 (PMC11498705; doi:10.1371/journal.pone.0312239)
Supplement: S3 Table — (DOCX) [file pone.0312239.s006.docx]

**S3 Table. The MM-GBSA analysis of the “V-TLR-2” and “V-TLR-4” complexes.**

| **Complex** | **VDW**  **(kcal/mol)** | **ELE**  **(kcal/mol)** | **GB**  **(kcal/mol)** | **SA**  **(kcal/mol)** | **Total binding energy**  **(kcal/mol)** |
| --- | --- | --- | --- | --- | --- |
| “V-TLR-2” | -346.7 | -860.9 | 1064.38 | -42.8 | -186.01 |
| “V-TLR-4” | -212.27 | -7131.08 | 7190.12 | -27.66 | -180.89 |
