# Supplementary material for: Designing novel multiepitope mRNA vaccine targeting Hendra virus (HeV): An integrative approach utilizing immunoinformatics, reverse vaccinology, and molecular dynamics simulation
Source: PLoS One. 2024 Oct 23;19(10):e0312239. doi: 10.1371/journal.pone.0312239 (PMC11498705; doi:10.1371/journal.pone.0312239)
Supplement: Attachment — Submitted filename: Response to Reviewers.docx [file pone.0312239.s008.docx]

Dear Respected Editors and Reviewers,

The time and effort you have devoted to reviewing our manuscript are greatly appreciated. The quality of our work has been significantly improved as a result of your insightful comments and suggestions.

Journal	Requirements
Please review your reference list to ensure that it is complete and correct. If you have cited papers that have been retracted, please include the rationale for doing so in the manuscript text, or remove these references and replace them with relevant current references. Any changes to the reference list should be mentioned in the rebuttal letter that accompanies your revised manuscript. If you need to cite a retracted article, indicate the article’s retracted status in the References list and also include a citation and full reference for the retraction notice.

**Ans.** We highly appreciate that you have mentioned this. We have verified each citation and reference and have substituted the retracted references with the appropriate ones. Furthermore, we have adjusted the citation style in the revised manuscript in accordance with the journal's specifications (PLoS). The file titled **Revised Manuscript with Track Changes** comprises all modifications, which are indicated in yellow.

**Additional Editor Comments:**

Please include a brief justification for the use of mRNA vaccines in both the discussion and conclusion to address the reviewer’s suggestion. Additionally, it is recommended to change the background of Figure 6 from black to white for better quality.

**Ans.** We have mentioned the brief justification for the use of mRNA vaccines in both the discussion and conclusion to address the reviewer’s suggestion.

Furthermore, in accordance with your recommendation, we have modified Figure 6. Now, it is displayed on a white background.

Additionally, we have improved the quality of all figures, corrected the table captions, figure captions, and images.
